# Global research trends in non-coding RNA and intestinal epithelial homeostasis: an integrated bibliometric analysis (2007–2024)

**DOI:** 10.1186/s41065-025-00560-y

**Published:** 2025-10-17

**Authors:** Xinxin Wang, Xuan Li, Yongtian Wen, Bochao Yuan, Xudong Tang, Ting Chen

**Affiliations:** 1 Institute of Digestive Diseases, China Academy of Chinese Medical Sciences Xiyuan Hospital, Beijing, 100091 China; 2 Graduate Institute of China Academy of Chinese Medical Sciences, Beijing, 100700 China; 3 China Academy of Chinese Medical Sciences Eye Hospital, Beijing, 100040 China; 4 China Academy of Chinese Medical Sciences, Beijing, China

**Keywords:** Non-coding RNA, Research trend, Bibliometrics, Intestinal barrier, MiRNA, CircRNA, LncRNA

## Abstract

**Background:**

Non-coding RNAs regulate gene expression through various mechanisms, playing key roles in life activities. Intestinal epithelial homeostasis (IEH) is a dynamic equilibrium state formed by the interaction of intestinal mucosal barrier, intestinal environment and other factors. There are many recorded documents demonstrating a close relationship between non-coding RNAs and IEH. Therefore, it is significant to retrospectively explore the current status, hotspots and trends of non-coding RNA (ncRNA) and IEH through a bibliometric perspective.

**Methods:**

We searched the Web of Science (WOS) for studies related to non-coding RNA and intestinal epithelial homeostasis from 1980 to December 2024, then downloaded the data. CiteSpace 6.3.1, VOSviewer 1.6.20, SCImago Graphica and Excel software were used to draw knowledge visualization maps.

**Results:**

A total of 667 publications relevant to ncRNA and IEH were included. The publication output and citation counts generally demonstrated an initial increase followed by a subsequent decrease. The publications have received a total of 18,979 citations, with an average of approximately 28 citations per article. China published the most articles, followed by the United States. Keyword analysis indicated that inflammatory bowel disease (IBD), intestinal immunity, and gut microbiota were of high importance. IBD dominates IEH research, yet circRNA is an emerging focus. Exploring ncRNAs in intestinal stem cell and colitis-associated cancer holds great research potential in the future. Multidisciplinary integration is the research trend.

**Conclusions:**

Our findings provide a comprehensive knowledge framework for researchers and clinicians aiming to explore ncRNA-based diagnostic and therapeutic strategies for IEH. Our results highlight the need for translational research to validate ncRNA-based diagnostic and therapeutic strategies for gut barrier disorders.

**Supplementary Information:**

The online version contains supplementary material available at 10.1186/s41065-025-00560-y.

## Introduction

Intestinal epithelial homeostasis (IEH) refers to the state in which intestinal epithelial cells maintain a dynamic balance in intestinal physiological functions and defense mechanisms. The intestinal epithelial cells, intestinal flora and immune system interact with each other to maintain IEH [1, 2]. Intestinal homeostasis is critical for nutrient absorption, immune defense, metabolic regulation, anti-aging and emotional regulation. Dysfunction of IEH can lead to a variety of diseases such as irritable bowel syndrome (IBS), inflammatory bowel disease (ulcerative colitis and Crohn’s disease) or bowel cancer, seriously affecting the quality of life of patients worldwide [3, 4].

Non-coding RNAs (ncRNAs) represent a class of functional RNA molecules that are not translated into proteins, mainly including microRNA (miRNA), long non-coding RNA (lncRNA), circular RNA (circRNA), etc. They play different roles in a wide variety of biological processes, such as epigenetic control of chromatin, promoter-specific gene regulation, macromolecular modification, regulation of mRNA stability, cell cycle regulation, etc [5, 6]. Non-coding RNAs widely influence intestinal immune homeostasis, intestinal inflammatory diseases and tumors. They influence the development of intestinal immune cells and regulate intestinal immune homeostasis through complex regulatory networks. In addition, they usually participate in the initiation and termination of intestinal inflammatory responses, as well as activation and inhibition of signaling pathways [7, 8].

The pathological disruption of IEH stems from the combined effects of immune dysregulation, inflammatory responses, and mucosal structural changes, manifesting as intestinal diseases such as IBS, IBD, and colitis - associated cancer (CAC) [9–12]. miRNAs, lncRNAs, and circRNAs can synergistically participate in the maintenance or disruption of IEH by regulating inflammation, barrier function, microbiota interactions, and key signaling pathways, thereby collectively influencing these related diseases. MicroRNA-155-5p inhibition alleviates IBS through Caudin-1 and ZO-1 expression [13]. In IBD, lncRNA CARINH maintains microbiota homeostasis by regulating antimicrobial protein families [14]. CircHIPK2 promotes TAZ protein translation through binding to EIF4A3, activates the Hippo signaling pathway, and CAC in the AOM/DSS model [15].

Bibliometrics, as an interdisciplinary subject, can make quantitative analysis of various knowledge carriers by using mathematical and statistical methods. The discipline is able to evaluate publishing trends, construct scientific knowledge maps and analyze topic clusters through graphical visualization [16, 17]. Therefore, bibliometrics serves as a vital analytical tool for evaluating academic research outputs, identifying emerging research trajectories, and assessing journal quality and influence through citation-based metrics. Its applications include evaluating institutional and researcher performance, enhancing knowledge integration across fields, and supporting strategic resource management in various academic areas.

Non-coding RNAs have emerged as critical regulators in intestinal diseases, especially in the regulation of intestinal immune homeostasis and the development of intestinal inflammatory diseases. Yet, a quantitative synthesis of ncRNA trends in IEH is lacking. Therefore, the paper makes an in-depth discussion on this topic from the perspective of bibliometrics and summarizes the latest progress and related research hotspots concerning ncRNAs and IEH.

## Materials and methods

### Data source and search strategy

The Web of Science Core Collection (WoSCC) database was used to extract literature data by adopting advanced retrieval strategy. The comprehensive literature search strategy was provided in the Supplementary Materials. The criteria for inclusion of English literature were as follows: (1) Type of literature: article or review; (2) Time span: January 1980 - December 30, 2024. Exclusion criteria: (1) dissertations, conference papers, newspapers, editorials, books, etc. (2) retracted publications and book Chaps. (3) duplicates, irrelevant topics.

**Fig. 1:**
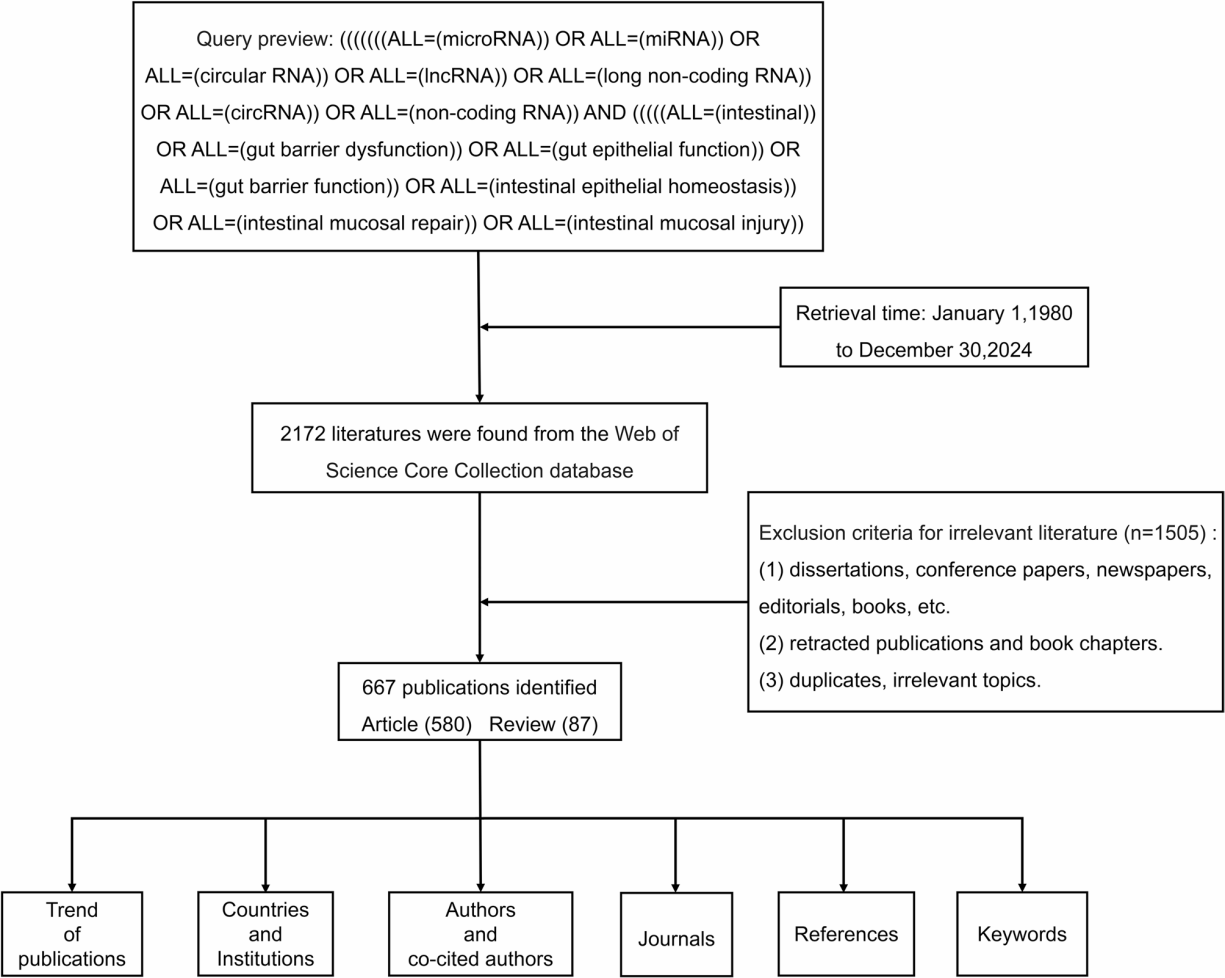
Flowchart of data search and analysis

We initially retrieved a total of 2172 articles. All retrievals were completed and downloaded on December 30, 2024 to avoid bias due to routine database updates. Two reviewers independently screened the title, abstract and citation of each article to exclude articles unrelated to ncRNAs and IEH. In cases of disagreement, consensus was achieved through discussion with a third screener. 2172 full-text papers were retrieved and read. After reading the full text, a total of 1505 articles were excluded and 667 articles mainly discussing ncRNA and IEH topics were included. For articles that met the requirements, we stored them as a plain text download_txt file (Fig. 1). Each text contained required analysis information such as title, author, keywords, abstract, research organization, and citation references.

### Software for data analysis

The study used Excel, VOSviewer 1.6.20, SCImago Graphica and CiteSpace 6.3.1 as the primary software tools for performing bibliometric analysis. Excel was used to conduct analysis of publication trend. VOSviewer and SCImago Graphica were used to visually analyze country and region cooperation. CiteSpace was used to visualize collaborations of institutions and authors. In addition, the overlay of dual graphs, keyword analysis, and citation burst of references in journals are all accomplished by CiteSpace.

CiteSpace, as a Java application, supports visual exploration with knowledge discovery in bibliographic databases. Through the progressive visualization method for knowledge domains, highly cited and key documents, specialized fields within knowledge areas, and the emergence of research topics can be intuitively mapped [18].

After importing all literature records into CiteSpace and removing duplicates, we applied the following parameters: (1) Time span: 2007–2024, year of each slice; (2) Selection thresholds: Top *N* = 50, Top N% = 10.0; (3) Maximum number of selected items: 100 per slice.

VOSviewer is an commonly used bibliometric software developed by professors van Eck and Waltman at Leiden University [19]. It can present the large-scale bibliometric maps to facilitate the visualization and clustering of literature from different countries and institutions [20].

Since the research data came directly from public databases and did not directly address the ethics of humans and animals, it did not require review by an institutional review committee.

## Results

### Analysis of publication outputs

According to our search strategy, over the past nearly two decades, a total of 667 articles, including 580 articles and 87 reviews, have been published on the study of IEH from the ncRNA perspective. Collectively, these publications have accrued a total of 18,979 citations, with an average of approximately 28 per article.

**Fig. 2:**
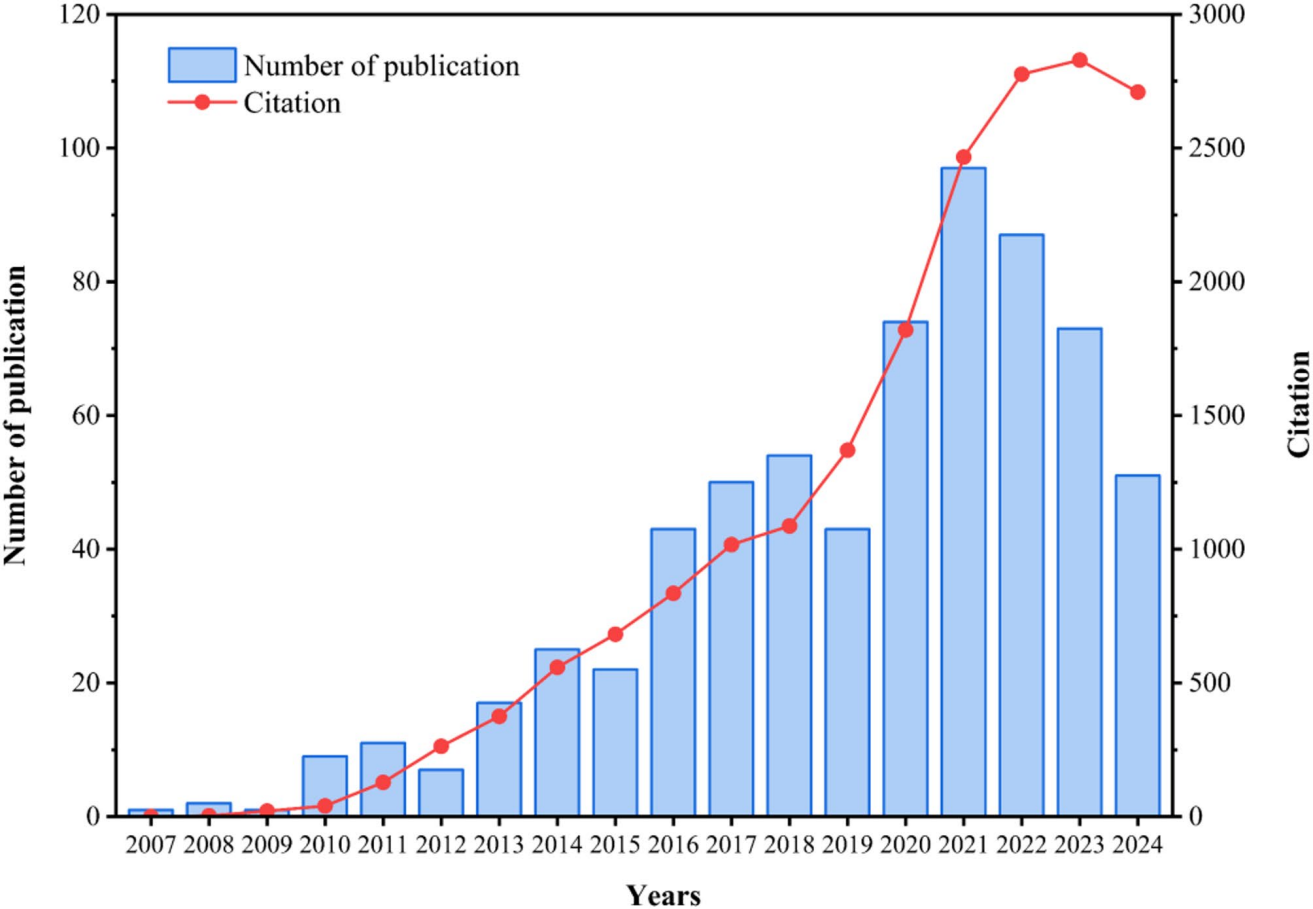
Global trends in the growth of publications and citations (2007–2024)

As shown in Fig. 2, the literature related to ncRNA and IEH gradually increased after 2007, while there were no papers on this topic before then. Prior to 2019, the number of the papers had been showing a gradual increase, despite minor declines every two to three years. From 2019 to 2021, the publication volume of papers on this topic surged rapidly, peaking at 97, followed by a significant reduction until 2024. This decline may be attributed to the global redirection of scientific resources toward pandemic-related studies after 2021, diverting funding and laboratory capacity from ncRNA-IEH research, alongside disrupted international collaborations and delayed experimental validation. The change of the number of citations of such literatures was the same as the trend of the number of publications, which gradually increased from 2007 and had a decreasing trend since 2023. Obviously, the research hotspot on ncRNAs and IEH has weakened in the past three years. Additionally, the overwhelming majority of publications mainly focus on molecular mechanisms, with few clinical studies identified. Regarding the scarcity of clinical evidence, conducting large-scale cohort research is imperative.

### Analysis of country (or region) Cooperation

**Fig. 3:**
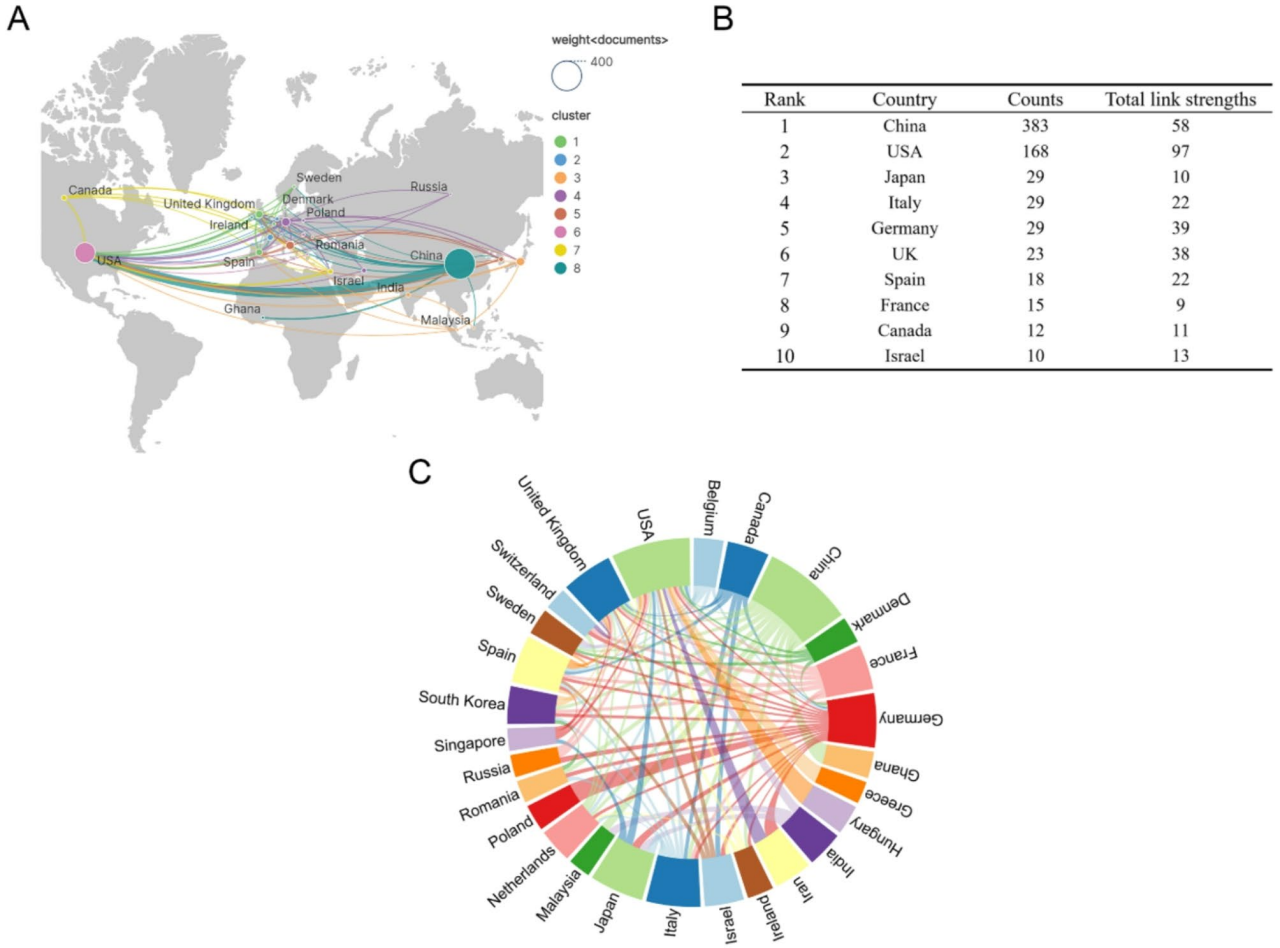
Publication distribution of countries. (**A**) Geographical co-occurrence network of countries and regions (**B**) Top 10 countries on research of ncRNAs in IEH (**C**) Chord diagram of national cooperative relations

The publications come from 53 countries. The top 10 countries are in Asia, North America and Europe. The size of the nodes and labels in the network is proportional to the number of publications, and the connections represent cooperation and closeness between institutions [21, 22]. The country with the largest number of publications is China (*n* = 383), followed by the USA (*n* = 168). We then filtered and visualized 53 countries with the criterion of having a publication count greater than or equal to 3, and constructed a network based on the number of publications and partnerships in each country (Fig. 3A, B).

It is noteworthy that there exists a significant amount of positive collaboration among various countries. USA engages in the most intensive cooperation with others. In addition, the cooperation between the USA and China is the closest. USA also maintains close cooperation with the UK, Germany and Israel (Fig. 3B, C). Crucially, the USA holds the highest centrality in the collaboration network, in contrast to China’s dominance in research output. The disparity arises from the USA’s longstanding commitment to facilitating transnational projects and resource sharing, which reinforces its role as a collaborative hub. China leads in publication output through substantial domestic funding support. The close USA-China cooperation underscores strategic complementarity, fostering knowledge integration across different scientific research paradigms.

### Analysis of institution Cooperation

To explore the research on ncRNA and IEH from different institutions around the world, we analyzed the number of publications from different institutions. Of the six institutions with the most publications in this field, four institutions are from China and two are from the USA.

**Fig. 4:**
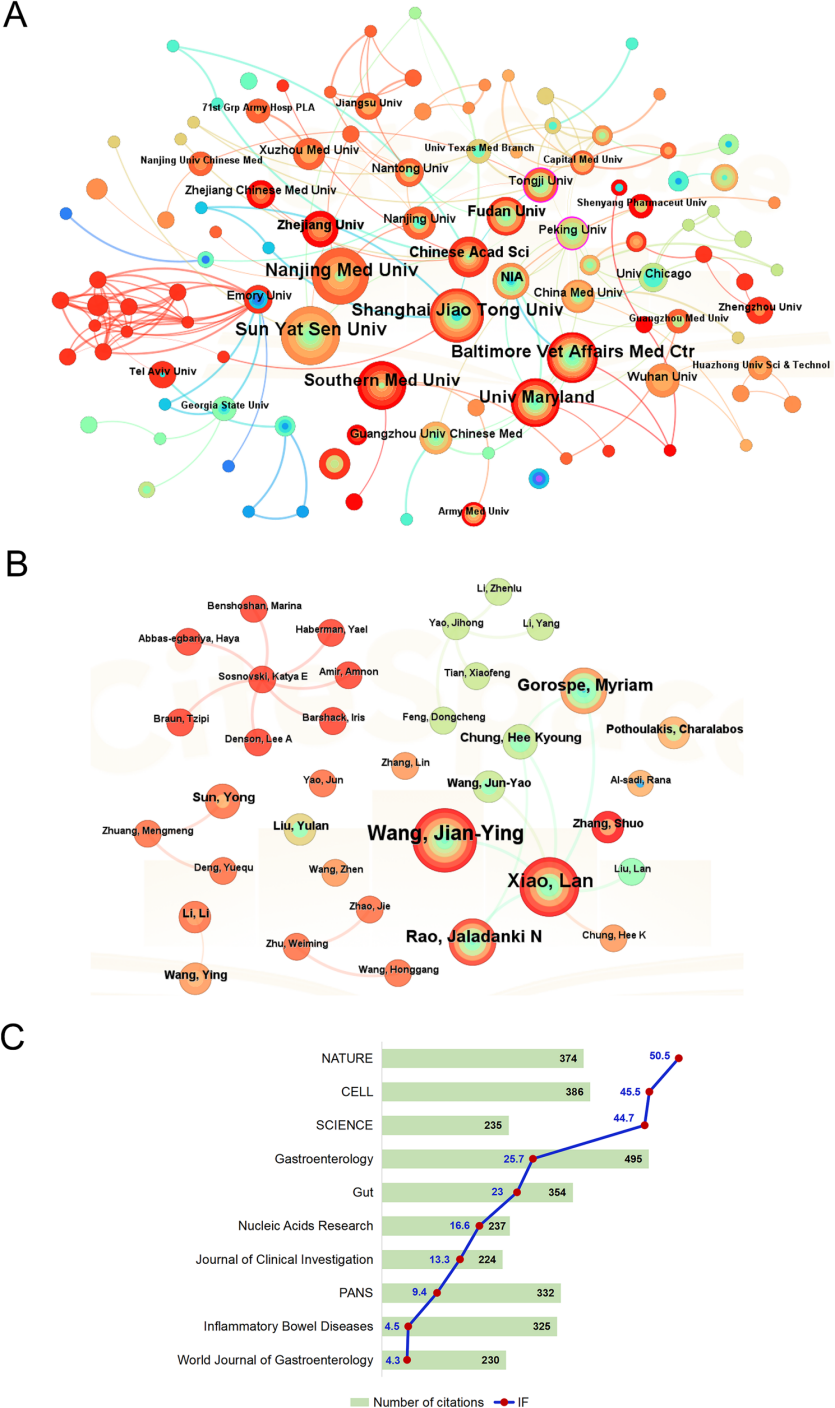
Analysis overview of institutions, authors and journals in ncRNA-IEH research. (**A**) Institutional collaboration network (**B**) Author collaboration network (**C**) Top 10 journals and their impact factor

Among the 296 institutions worldwide, Sun Yat-Sen University (25), Nanjing Medical University (23) and Baltimore Vet Affairs Medical Center (22) ranked among the top three institutions in the world in terms of publication volume (Table 1). We have established a collaborative network by analyzing the quantity and relationships of publications from each institution (Fig. 4A). In this institution cooperation network diagram, when the area of a node is larger, it means that the institution publishes more literature. The thicker the curve of the connecting nodes, the higher the frequency of their co-occurrence and the closer the cooperative relationship. Emory University, Chinese Academy of Sciences, Tongji University and Peking university and other different institutions have many close collaborations in this field. National Institute on Aging (NIA) has extensive collaborations with Peking University, University of Maryland and Baltimore Vet Affairs Medical Center. It is worth noting that only Sun Yat-Sen University and Baltimore Vet Affairs Medical Center have published articles annually without intervals in the past 14 years. Furthermore, the earliest institution to begin to study in the field of ncRNAs and IEH is the Baltimore Vet Affairs Medical Center, which has been engaged in this field for 14 years, and is also the longest institution to conduct research in this field.

**Table 1:** The top 10 productive institutions

Rank	Publications	Begin	End	Institutions
1	25	2016	2021	Sun Yat-Sen University*
2	23	2016	2022	Nanjing Medical University
3	22	2011	2024	Baltimore Vet Affairs Medical Center*
4	20	2012	2023	Shanghai Jiao Tong University
5	18	2017	2024	Southern Medical University
6	18	2012	2024	University of Maryland
7	13	2014	2020	Central South University
8	12	2014	2023	Chinese Academy of Sciences
9	11	2015	2023	Fudan University
10	10	2016	2022	Dalian Medical University

* indicates that this institution publishes articles every year without intervals

### Analysis of author and cited author

Citespace was used to analyze the authors of literatures and map the cooperation network. Professor Wang Jianying of the University of Maryland ranked first with 17 papers, followed by Professor Xiao Lan of Baltimore Vet Affairs Medical Center with 15 articles. In addition, Professor Rao Jaladanki N of the University of Maryland published 10 articles (Table 2). These prolific scholars worked closely with each other (Fig. 4B). Other academics with fewer publications seem to need more in-depth collaboration.

Among the cited papers, Professor Wu Feng’s 16 papers on ncRNA and IEH were frequently cited 152 times (Table 3). More importantly, if a node is highly intermediated in the network, it is more important to connect different nodes [23]. Wu Feng had the highest centrality of 0.63, indicating that this scholar has a very important influence on the transmission of information and the connectivity of the network. It can be seen that Wang Jianying and Wu Feng has done in-depth research in the field of ncRNA and IEH, which has a certain authority and influence. Kalla R achieved exceptional impact with 54 citations from a single paper (Table 3). As his work has made foundational contributions to the field of ncRNA and IEH, he remains highly cited despite lower publication output.

**Table 2:** The 5 most productive authors

Rank	Frequency	Name
1	17	Wang Jian-Ying
2	15	Xiao Lan
3	10	Rao, Jaladanki N
4	9	Gorospe, Myriam
5	5	Sun Yong

**Table 3:** The top 10 authors cited by others

Rank	Author	Centrality	Document	Citation	Citation/Document
1	WU F	0.63	16	152	9.50
2	OCONNELL RM	0.25	8	50	6.25
3	XIAO L	0.24	10	60	6.00
4	MCKENNA LB	0.16	4	60	15.00
5	BARTEL DP	0.15	14	121	8.64
6	ZHANG L	0.06	4	65	16.25
7	ZHANG Y	0.04	3	62	20.67
8	YE DM	0.04	5	59	11.80
9	KALLA R	0	1	54	54.00
10	LI Y	0	1	46	46.00

### Analysis of journals and cited journals

Studies on ncRNAs and IEH were published in 287 academic journals from 2007 to 2024. The top 10 most cited journals and their impact factors are shown in Fig. 4C.

*Gastroenterology* ranked first with 495 citations, followed by *Cell* at second with 386 citations and *Nature* at third with 374 citations. After analyzing the top 10 academic journals in terms of citations, we found that seven journals were from the USA and three were from the UK. At the same time, these journals with a high volume of publications have high impact factors, and they have played a good leading role in the research of ncRNA and IEH. These journals were widely recognized for their tremendous influence in the study of ncRNA and IEH.

**Fig. 5:**
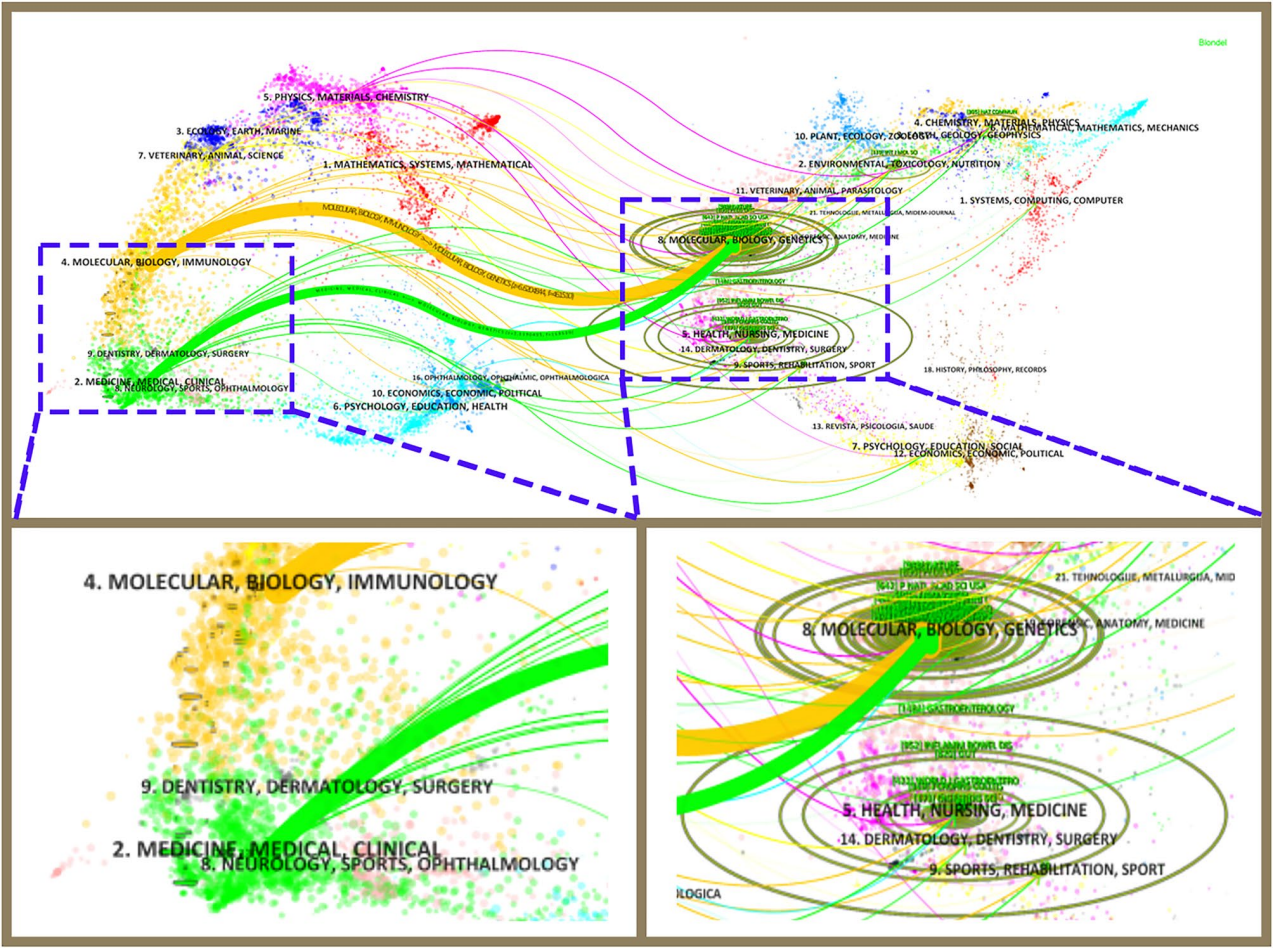
Dual-map overlay of the journals on ncRNA and IEH (2007–2024)

The dual-map overlay of journals was a method to display the distribution of citation relationships between journals, with the cluster of citing journals on the left and the cluster of cited journals on the right. Orange and green reference path respectively shown the articles published in Molecular/Biology/Genetics journal literatures were cited respectively by Molecular/Biology/Immunology and Medicine/Medical/Clinical. (Fig. 5).

### Analysis of co-cited references

By analyzing the references, we can determine the change process of highly cited references over time, as well as how the research focus has changed over time.

Of the top 10 co-cited references (Table 4), “MicroRNAs: new players in IBD” (Kalla R, et al., 2015), published in *Gut* [24], has high credibility and academic value as the literature with the highest cited times (28 times). It has introduced the roles of miRNA in different aspects in IBD, including the developmental and regulatory roles in the innate and adaptive immune systems, autophagy, the integrity of the epithelial barrier and the clinical translational study of miRNA. “MicroRNA-31 Reduces Inflammatory Signaling and Promotes Regeneration in Colon Epithelium, and Delivery of Mimics in Microspheres Reduces Colitis in Mice” (Tian Y, et al., 2019) was published in *Gastroenterology* (26 times) [25]. Tian et al. discovered that miR-31 was increased in the colonic tissues of patients with IBD and also regulated the WNT and Hippo signaling pathways, promoting epithelial regeneration after injury. “MicroRNA 301A Promotes Intestinal Inflammation and Colitis-Associated Cancer Development by Inhibiting BTG1” (He C, et al.) was also published in *Gastroenterology* (21 times) [26]. Notably, the top 10 co-cited articles were published at least 5 years ago, and these articles have been reviewed and cited for many years, indicating that they were of high quality and academic value in the field of ncRNAs and IEH research.

**Fig. 6:**
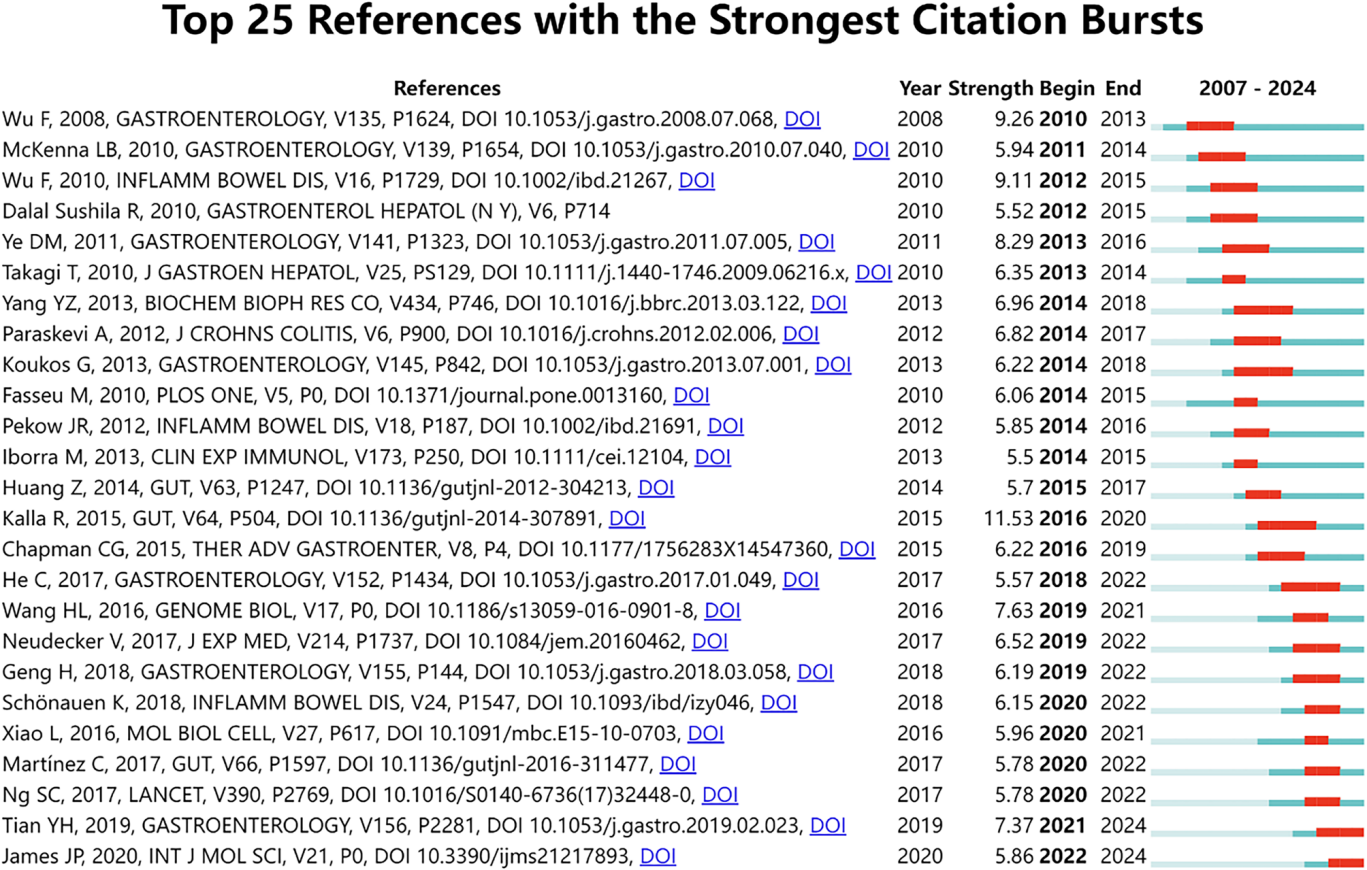
Top 25 references with strong citation bursts in ncRNA and IEH

The citation burst strength plot of references shows those references that have been cited frequently by other scholars in the fields of ncRNA and IEH over time. As shown in Fig. 6, the red bars represent the time periods in which citations burst directly from 2007 to 2024. We can see bursts of references on this topic being cited as early as 2008 and as late as 2020. The article title of the strongest citation burst was “MicroRNAs: new players in IBD” (strength = 11.53) published in *Gut*, and was also the most widely used literature, with a citation burst from 2016 to 2022.

The reference with the second strongest citation outbreak was “MicroRNAs are differentially expressed in ulcerative colitis and alter expression of macrophage inflammatory Pepti-2 alpha” (strength = 9.26) [27], published in *Gastroenterology* by Wu Feng and other scholars. The citation outbreak time was 2010 to 2013. Overall, the citation burst strength of the 25 references ranged from 5.5 to 11.53 and the durations varied from two to five years.

**Table 4:** Top 10 co-cited references in ncRNA and IEH

Rank	References	Journal	Co-citation	Publishing year
1	MicroRNAs: new players in IBD [24]	GUT	28	2015
2	MicroRNA-31 Reduces Inflammatory Signaling and Promotes Regeneration in Colon Epithelium, and Delivery of Mimics in Microspheres Reduces Colitis in Mice [25]	GASTROENTEROLOGY	26	2019
3	MicroRNA 301 A Promotes Intestinal Inflammation and Colitis-Associated Cancer Development by Inhibiting BTG1 [26]	GASTROENTEROLOGY	21	2017
4	Myeloid-derived miR-223 regulates intestinal inflammation via repression of the NLRP3 inflammasome [28]	J EXP MED	20	2017
5	Overexpression of miR-21 in patients with ulcerative colitis impairs intestinal epithelial barrier function through targeting the Rho GTPase RhoB [29]	BIOCHEM BIOPH RES CO	19	2013
6	In Inflamed Intestinal Tissues and Epithelial Cells, Interleukin 22 Signaling Increases Expression of H19 Long Noncoding RNA, Which Promotes Mucosal Regeneration [30]	GASTROENTEROLOGY	19	2018
7	Identification of microRNAs associated with ileal and colonic Crohn’s disease [31]	INFLAMM BOWEL DIS	18	2010
8	Pro-inflammatory miR-223 mediates the cross-talk between the IL23 pathway and the intestinal barrier in inflammatory bowel disease [32]	GENOME BIOL	18	2016
9	MicroRNA regulation of intestinal epithelial tight junction permeability [33]	GASTROENTEROLOGY	18	2011
10	MicroRNA-124 regulates STAT3 expression and is down-regulated in colon tissues of pediatric patients with ulcerative colitis [34]	GASTROENTEROLOGY	17	2013

**Fig. 7:**
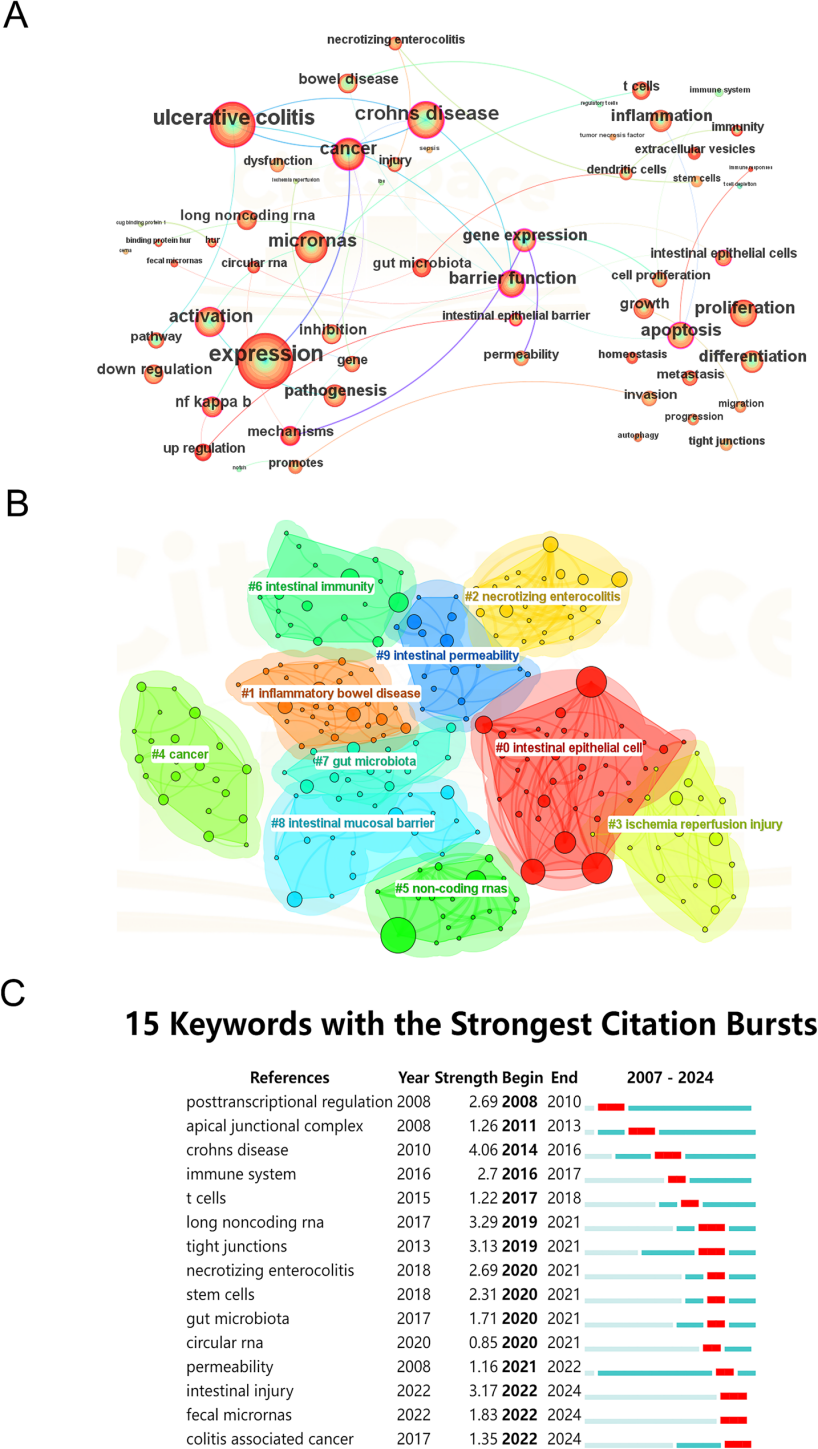
Keywords analysis overview in ncRNA and IEH research. (**A**) Collaborative network (**B**) Cluster results (**C**) 15 keywords with the strongest citation bursts

**Fig. 8:**
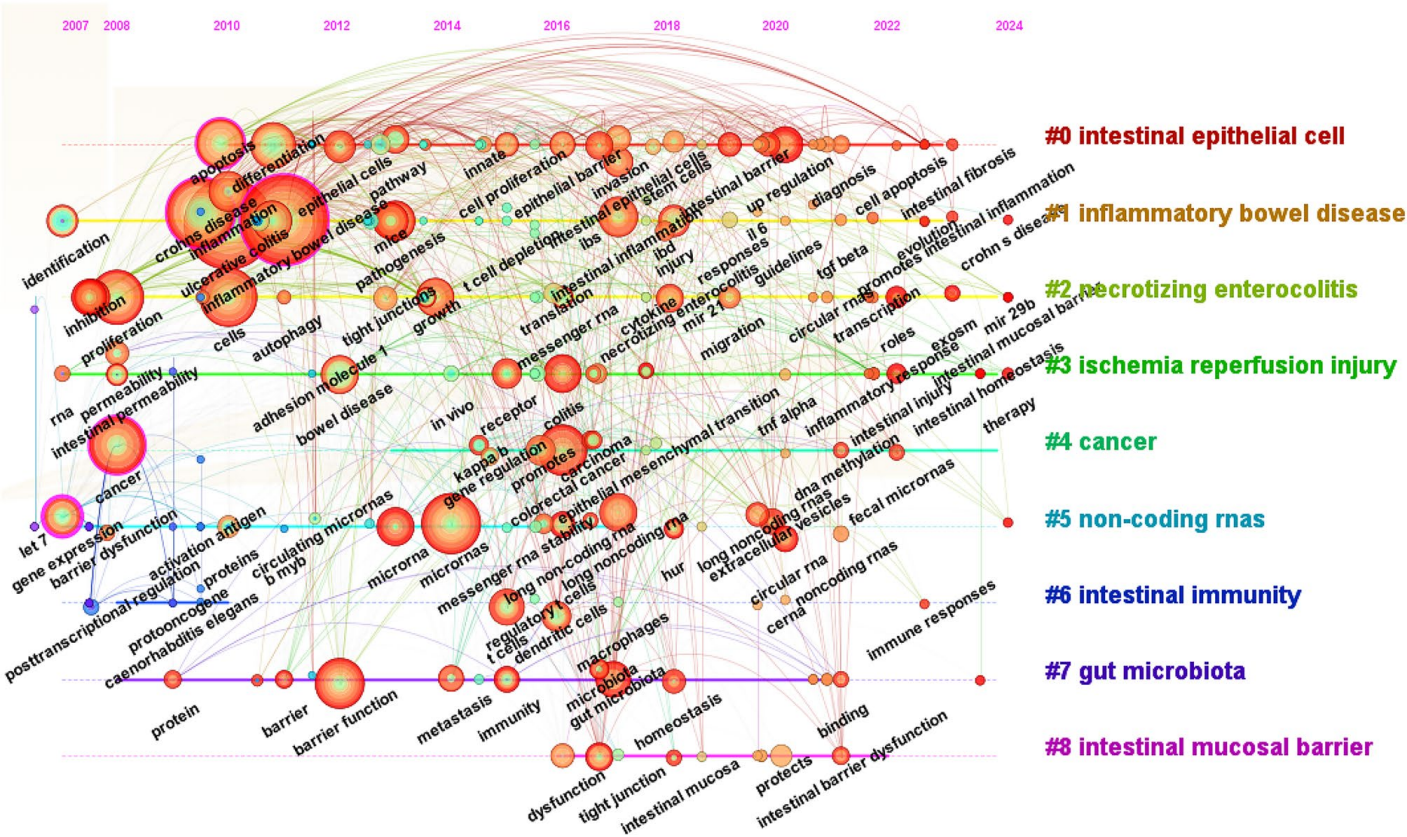
Timeline view of keywords in ncRNA and IEH research

### Analysis of keyword co-occurrence and burst keywords

Keyword analysis is the important content of bibliometrics, which is helpful for a comprehensive understanding of the research content of ncRNA and IEH. The analysis could briefly summarize the research topics of the literature and show the classification and evolution of literature research hotspots. Figure. 7A shows the keyword contribution network for ncRNA and IEH studies. The whole network has a total of 298 nodes, and 1144 links. The topology density of the network is 0.0259.

The larger the area of nodes in the network, the more attention is paid to the keyword. Among these keywords, “ulcerative colitis”, “crohns disease”, “crohns disease”, “cancer” and “micrornas” all appear more than 75 times, which represent the major research directions in the field of ncRNA and IEH.

In addition, through the cluster analysis of keywords (Fig. 7B), we can see the keyword clusters more clearly. The Q value of the keyword cluster was 0.4559 (Q > 0.3), and the S value was 0.7025 (S > 0.5). Each color represents an identical cluster group. Small node represents a keyword. The 10 clusters shown in Figs. 7B and 8 are as follows: #0 intestinal epithelial cell, #1 inflammatory bowel disease, #2 necrotizing enterocolitis, #3 ischemia injury, #4 cancer, #5 non-coding rnas, #6 intestinal immunity, #7 gut microbiota, #8 intestinal barrier, #9 intestinal permeability.

The burst strength analysis of keywords can better reflect the variation trend of some important keywords over time, so as to determine the future research direction (Fig. 7C). Obviously, the key word research hotspot was still “intestinal barrier” (strength = 1.16) in the last four years. “fecal microRNAs” intertwines with “colitis - associated cancer”, as fecal miRNAs serve as non-invasive biomarkers for CAC. This may represent a potentially promising future research direction. “circular RNA” and “stem cells” demonstrate co-occurring citation bursts, revealing circRNAs as “novel epigenetic modulators” that govern stem cell self-renewal and differentiation. “Crohns disease” had the highest intensity of outbreak keywords (strength = 4.84), indicating that Crohns disease has been a hot topic in the field of IEH research from 2011 to 2016. In this field, scholars once paid attention to the “immune system” (strength = 2.7), “gut microbiota” (strength = 1.71), “permeability” (strength = 1.16). They used these pathways to explore the effects of ncRNA on IEH. In addition, we can find that scholars studied IEH from the perspective of “long noncoding rnas” (strength = 4.84) from 2019 to 2021. The commonly studied miRNAs and lncRNAs were listed in Tables 5 and 6. Non-coding RNAs primarily regulate IEH through three core mechanisms: (1) miRNA-mediated post-transcriptional silencing, (2) lncRNA and circRNA competitively bind to miRNAs through the ceRNA (competing endogenous RNA) network, (3) direct pathway modulation. Most miRNAs act via translational repression, while lncRNAs and circRNAs dominate signaling and epigenetic interventions to coordinately regulate barrier integrity, inflammation, and cell fate.

**Table 5:** MicroRNAs in intestinal epithelial homeostasis (studied in ≥ 6 articles)

MicroRNA	Disease	Mechanism	Proposed Function
miR-31	sepsis	Inhibition of miR-31 decreased intestinal mucosal permeability and intestinal barrier function [67].	Impairs intestinal barrier function
	UC	MiR-31 can directly block TSLP from promoting mucosal healing and regulating inflammation [68].	Inhibits mucosal repair
	CD	MiR-31 directly regulated IL-25 expression and affect the mucosal IL-12/23-mediated Th1/Th17 pathway [69].	Affect Th1/Th17 pathway
miR-155	UC	MiR-155 appears to play a role in the intestinal inflammation of patients with active UC by downregulating the expression of FOXO3a [70].	Promotes inflammation
	CD	MiR-155 directly targets HBP1 to induce CD-associated intestinal fibrosis via Wnt/beta-catenin signalling pathway [71].	Promotes intestinal fibrosis
	NEC	The increase in intestinal inflammation and the activation of nuclear factor-kappa B were accompanied by a decrease in the expression of miR-155 in IEC [72].	Regulates inflammation
	sepsis	MiR-155 inhibitor alleviates inflammation and intestinal barrier dysfunction by inactivating NF-kappa B signaling during sepsis [73].	Inhibits inflammation & barrier dysfunction
miR-223	CAC	Overexpression of miR-223 inhibited Akt phosphorylation and IGF-1R expression in these cells [74].	Inhibits Akt/IGF-1R signaling
	IBD	MiR-223 could inducing intestinal barrier dysfunction through the inhibition of TMIGD1 [75].	Impairs barrier function
	IBD	MiR-223 limits intestinal inflammation by constraining the NLRP3 inflammasome [28].	Limits inflammation
miR-29a	UC	MiR-29a regulated intestinal epithelial apoptosis by down-regulating the expression of Mcl-1 [76].	Promotes apoptosis
	UC	MiR-29a inhibits rapamycin-induced intestinal epithelial cells’ autophagy partly by decreasing ATG9A in UC [77].	Inhibits autophagy
	IBS	MiR-29a effects on intestinal membrane permeability may be due to its regulation of GLUL [78].	Affects permeability
	IBS	MiR-29a increased the intestinal membrane permeability of colonic epithelial cells by reducing the AQPs expression in IBS-D rats [79].	Increases permeability
miR-195	/	MiR-195 disrupts Tuft cell function by inhibiting DCLK1 translation via interaction with HuR [80].	Disrupts Tuft cell function
	acute injury	MiR-195 regulates intestinal epithelial restitution after wounding by altering actin-related protein-2 translation [81].	Regulates epithelial restitution
	sepsis	MiR-195 regulates SIRT1-mediated downstream effectors in ER stress-induced apoptosis in sepsis [82].	Regulates apoptosis
miR-29b	CD	Overexpression of miR-29b in CD fibroblasts led to a down-regulation of collagen I and III transcripts and collagen III protein [83].	Inhibits collagen production
	/	MiR-29b inhibits expression of LRP6 and HuR post-transcriptionally, thus playing a role in the regulation of IEC proliferation and intestinal epithelial homoeostasis [84].	Regulates IEC proliferation & homeostasis
	/	MiR-29b silencing prevented JunD-induced repression of IEC proliferation [85].	Affects IEC proliferation
miR-146a	UC	MiR-146a restricts the expansion of intestinal T cell populations, including Th17, Tregs, and Tfh cells [86].	Restricts T cell expansion
	intestinal I/R injury	Induction of miR-146a by the phytochemical diindolylmethane controlled Irak1 upregulation and prevented immune hyper-responsiveness [87].	Prevents immune hyper-responsiveness
miR-142-3p	IBD	Insufficient vitamin D levels alter expression of autophagy-regulating miR-142-3p in intestinal tissues of patients with IBD [88].	Autophagy
miR-21	IBD	MiR-21 regulate NF-kappa B or mTOR signaling to induce or inhibit autophagy in intestinal cells by releasing anti-or proinflammatory factors [89].	Modulates autophagy & inflammation
	IBD	MiR-21 deletion exacerbated CD4(+) T-cell-mediated models of colitis provide further evidence that miRNAs play significant roles [90].	Modulates immunity
	CD	MiR-21 inhibition relieved intestinal fibrosis by regulating extracellular matrix (ECM) re-modeling [91].	Promotes fibrosis
	UC	MiR-21 may regulate intestinal epithelial tight junction permeability through PTEN/PI3K/Akt signalling pathway [92].	Regulates tight junction
miR-16	IBD	Inhibiting expression of miR-16 significantly decreased the expression of interleukin-1 beta, interleukin-6, and tumor necrosis factor-alpha [93].	Inhibits inflammatory cytokines
	IBS	MiR-16 down-regulates JIST through the TLR4/NF-KB pathway, thereby relieving IBS-D [94].	Downregulates JIST
	IBS	MiR-16 is involved in barrier function dysregulation through the modulation of claudin-2 and cingulin expression in the jejunum in IBS [95].	Alters barrier dysregulation
miR-145	intestinal I/R injury	MiR-145 negatively regulates the expression and function of P-gp through the repression of mRNA by direct interaction on the 3’-UTR of MDR1 mRNA [96].	Negatively regulates P-gp function
miR-143	CD	MiR-143 may induce bowel inflammation by regulating ATG2B and autophagy [97].	Autophagy

**Table 6:** LncRNAs in intestinal epithelial homeostasis (studied in ≥ 3 articles)

LncRNA	Disease	Mechanism	Proposed Function
DANCR	sepsis	DANCR improved intestinal barrier dysfunction and alleviated epithelial injury by targeting the miR-1306-5p/PLK1 axis [98].	Protects barrier function
H19	UC	LncRNA-DANCR reduced inflammation, cell apoptosis and up-regulated ZO-1, MUC2 and Claudin-1 [99].	Reduces inflammation
	UC	LncRNA-H19 was highly expressed in UC mice and bound to miR-331-3p to promote TRAF4 transcription, thereby aggravating intestinal injury [100].	Promotes injury
	severe burn	LncRNA H19 may regulate the repair of EGF via let-7 g following intestinal mucosa injury after a burn [101].	Regulates mucosal repair
ANRIL	CD	LncRNA ANRIL downregulation in intestinal mucosa correlates with increased disease risk, higher disease activity and elevated proinflammatory cytokines levels [102].	Correlates with disease severity
Lnc13	Celiac Disease	Increased peripheral expression of lnc13 along with their decreased duodenal expression demonstrates CeD [103].	Diagnostic marker
SPRY4-IT1	septic stress	SPRY4-IT1 silencing led to dysfunction of the epithelial barrier in cultured cells by decreasing the stability of mRNAs encoding TJ proteins claudin-1, claudin-3, occludin, and JAM-1 and repressing their translation [104].	Maintains barrier integrity
MEG3	sepsis	MEG3 was decreased whereas miR-129-5p was obviously increased in Caco2 cells incubated with LPS [105].	Modulates inflammation
	intestinal I/R injury	MEG3 also activated the expression of sirtuin 1 (SIRT1) by Caco-2 cells via sponging miR-34a-3p and relieved CA-induced intestinal barrier dysfunction through NF-kB signaling pathway [106].	Protects barrier function
MIR4435-2HG	UC	MIR4435-2HG suppression inhibits macrophage M1 polarization while promoting M2 polarization, thereby alleviating intestinal inflammation through JAK1/STAT1 signaling pathway [107].	Regulates macrophage polarization
uc.173	/	Lico A promoted the proliferation and inhibited the apoptosis of IECs through uc.173/miR-195 pathway [108].	Promotes IEC proliferation
	septic stress	uc.173 in controlling gut permeability and define a mechanism by which uc.173 stimulates claudin-1 translation, by decreasing the availability of miR-29b to CLDN1 mRNA [109].	Maintains gut permeability
	IBS	The down-regulation of LncRNA H19 resulted in the expression changes of AQP1 and AQP3 may play an important role in the development of IBS-D [110].	Regulates the expression of AQP1
MALAT1	/	Knockdown of MALAT1 downregulated SOX9 expression by binding to miR-129-5p, thereby inhibiting the abnormal proliferation of IECs via the WNT/beta-catenin signaling pathway [111].	Regulates IEC proliferation
	CD	MALAT1 regulated the intestinal mucosal barrier and regained intestinal homeostasis by sequestering miR-146b-5p and maintaining the expression of the apical junction complex proteins NUMB and CLDN11 [112].	Preserves mucosal barrier
NEAT1	UC	Down-regulating NEAT1 inhibited the activity of ROCK/MLCK signaling, reducing inflammatory response, thereby repairing the intestinal barrier function [113].	Regulates barrier function
	UC	Silencing NEAT1 effectively ameliorates the LPS-induced IECs dysfunction [114].	Regulates IECs dysfunction
GAS5	Celiac Disease	The increase in NEAT1 expression after gluten exposure was mediated by IL-15 and STAT3 activation and binding to the NEAT1 promoter [115].	Mediates gluten response
	IBD	Downregulation of NEAT1 suppressed the inflammatory response by modulating the intestinal epithelial barrier and through exosome-mediated polarization of macrophages in IBD [116].	Suppresses inflammation
	IBD	The expression of GAS5 was also determined in the human monocytic THP1 cells differentiated into macrophages and stimulated with lipopolysaccharide (LPS) [117].	Regulates Inflammatory response
	IBD	GAS5 is involved in regulating intestinal MMP-2 and MMP-9 in pediatric patients with IBD [118].	Regulates MMPs
Nostrill	/	Nostrill enhances the transcription of a set of genes regulated by IFN-gamma in intestinal epithelial cells [119].	Enhances IFN-γ response
	/	Induction of Nostrill in infected intestinal epithelial cells was triggered by NF-kappa B signaling and was observed to enhance epithelial defense by decreasing parasitic infection burden [120].	Strengthens epithelial defense
TUG1	UC	TUG1 inhibits IEC apoptosis and UC progression by regulating the balance of HuR and miR-29b-3p [121].	Inhibits apoptosis
	HGHF	TUG1 alleviated the damage induced by HGHF in intestinal epithelial cells by downregulating SIRT1 and AMPK expression, and upregulating UCP2 expression [122].	Alleviates cellular damage

**Fig. 9:**
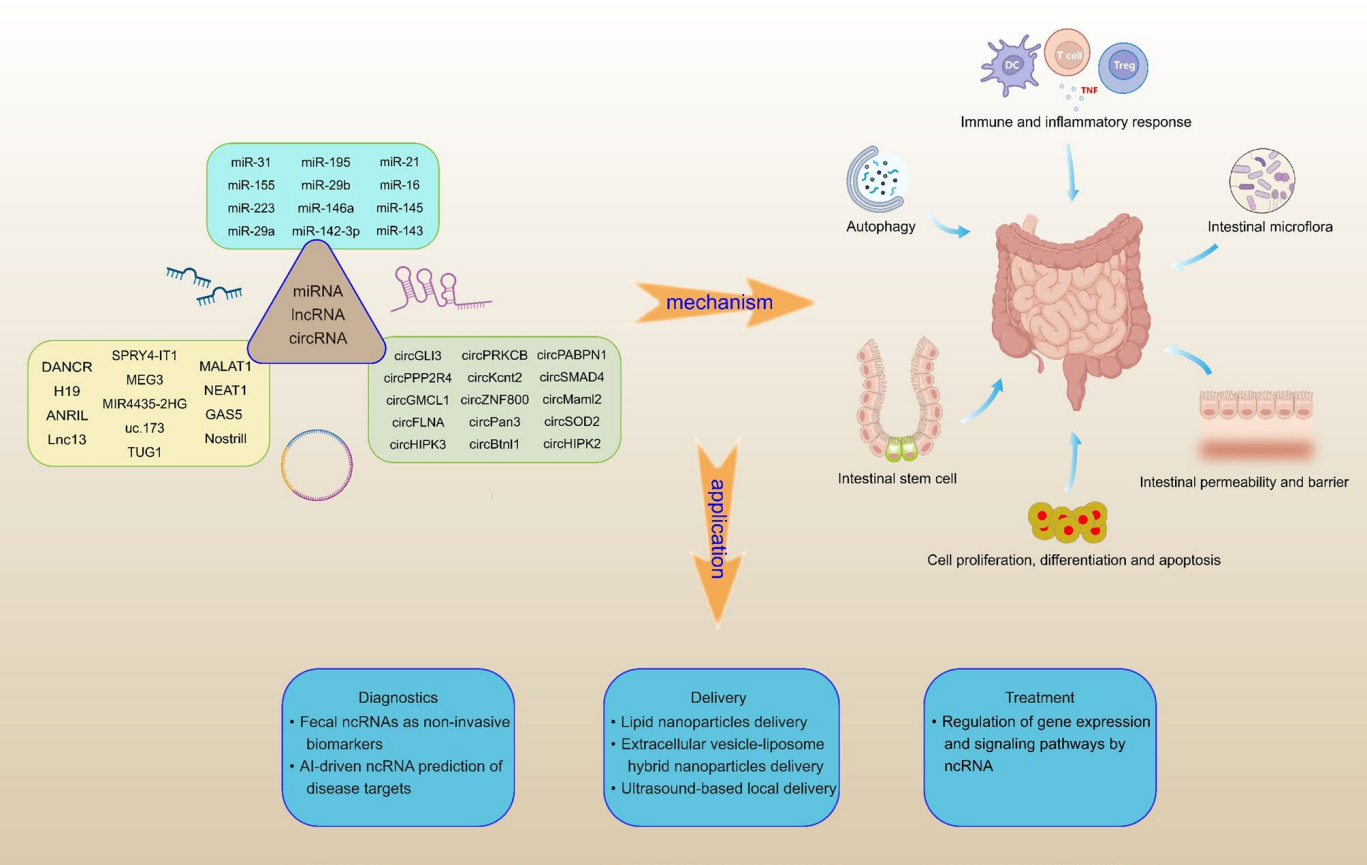
Framework for ncRNA-mediated regulation of IEH and application roadmap

MiRNA, lncRNA, and circRNA regulate IEH through different mechanisms (Fig. 9). The regulation of ncRNA can promote intestinal mucosal repair and intestinal barrier construction. These ncRNAs exert critical regulatory functions in UC, CD, IBS, intestinal ischemia-reperfusion (I/R) injury, colonic-inflammation-induced carcinogenesis, necrotizing enterocolitis, celiac disease and other intestinal diseases, which are also ideal disease models for conducting IEH research. These ncRNAs mainly regulate IEH through the following mechanisms: (1) regulating the inflammatory response (ANRIL, miR-223, miR-155, NEAT1, H19) (2) affecting intestinal permeability (miR-29a, MEG3, miR-31, uc.173, miR-31) (3) regulating intestinal immune cells (miR-146a, MIR4435-2HG) (4) regulating intestinal autophagy (miR-21, miR-142, miR-29a) (5) affecting the function and repair of the intestinal epithelial barrier (miR-29b, miR-195, MALAT1, DANCR, miR-16, TUG1) (6) regulating the intestinal microenvironment (NOSTRILL).

## Discussion

In the past two decades, with the development of high-throughput sequencing technologies, researchers have discovered more ncRNAs and gained a deeper understanding of their functions by analyzing the entire transcriptome. Non-coding RNAs regulate gene expression through a variety of mechanisms and exerted pivotal regulatory influences on the regulation of various aspects of life activities [35]. The study of ncRNAs has not only expanded the understanding of gene regulation, but also has far-reaching impacts in the fields of cancer [36] and neurological disorders [37], providing new ideas for the diagnosis and treatment of diseases [38]. IEH refers to the state in which intestinal epithelial cells maintain structural and functional balance during continuous renewal. Currently, the dysregulation of IEH and the molecular mechanisms for repairing its integrity have not been clarified, which hampers the proper diagnosis and accurate treatment of these diseases.

Non-coding RNAs exert significant impacts on regulating IEH. The study provided the first detailed analysis of the major knowledge areas and emerging trends in the literature on ncRNAs and IEH. We used novel knowledge aggregation methods and advanced bibliometric techniques to visualize this research area and analyzed the scholars, research institutions, and countries that have contributed most to the field. Furthermore, this study evaluated the past research hotspots in this field, and the evolution of future research directions.

### Global research trends on the regulation of IEH by ncRNAs

As shown in Fig. 2, the rise of related research after 2007 may be due to the popularization and development of sequencing technology and molecular biology research methods at that time, which made it possible to study IEH from the perspective of ncRNAs. Since then, the annual number of publications and citations in this field has grown steadily. This trend was probably due to increased research funding, which attracted more researchers and led to a significant increase in research output. However, there was a significant decline in 2021, perhaps because the research had entered a bottleneck, and it was difficult to make further breakthroughs in some key issues.

Although the number of publications and citations reflect the research hotspot to a certain extent, they cannot be fully equated with the research quality. During the change of research hotspot in this field, we should focus on the control of research quality. Even in the period of declining interest, there may be high-quality and innovative research results that are overlooked. In fact, the decline in publications may not stem from scholars abandoning the theme of IEH, but rather from waning novelty and research enthusiasm for ncRNAs. However, it is known that the 2024 Nobel Prize in Physiology or Medicine was awarded to American scientists Victor Ambros and Gary Ruvkun for their discovery of miRNAs and their key role in post-transcriptional gene regulation [39]. This contemporary recognition may further stimulate investigation into ncRNA-mediated mechanisms of IEH.

The analysis of countries and institutions shows China leads in publications and has established extensive international collaborations. The United States, while ranking second in output, maintains the largest number of collaborative partnerships globally. Figures 3 and 4A show that although there are now collaborations between different institutions around the world, we can further explore more diversified modes of cooperation, such as international multi-center joint research projects, to integrate global resources and improve research efficiency and quality of results. In addition to focusing on the top-tier institutions with rich achievements, such as the Baltimore VA Medical Center and the University of Maryland, attention should also be paid to emerging institutions with relatively low publication outputs but rapid growth or unique insights in specific research directions. Providing them with great support and collaboration opportunities can foster innovation and drive breakthroughs in the field. In addition, the analysis of authors’ publications, collaborative networks and citations clearly presented the distribution of academic power in the field. The close collaboration among high-productivity authors, such as Wang Jianying, Xiao Lan and Rao, Jaladanki N, was conducive to integrating resources and promoting research progress, while the insufficient output and influence of the research results of low-productivity authors may be due to the limitation of their collaboration. Furthermore, we found that while U.S. research institutions ranked first in publication output, it was mainly Chinese scientists in the U.S. who contributed to this field, such as Prof. Jian-Ying Wang at the University of Maryland and Prof. Feng Wu at Johns Hopkins University Healthcare, suggesting that transnational mobility of talent greatly contributed to knowledge innovation in this field.

### Multidisciplinary integration is the future research trend in this field

In the process of citation analysis of journals, we found that the research on ncRNA and IEH involves multiple disciplines, such as molecular biology, pharmacy, immunology, and clinical medicine. Joint research carried out by authors with different disciplinary backgrounds can introduce new research perspectives and methods, giving rise to more innovative achievements. We believe that in the future, the research on ncRNA and IEH can be integrated with more disciplines such as artificial intelligence, bioinformatics, and material science. The GCNCMI model for miRNA target prediction and lipid nanoparticles for ncRNA delivery have achieved the integration of artificial intelligence, materials science, and medicine [40]. It was reported that the team of Hailong Zhang found that NLRP3 siRNA delivered by cationic liposomes for the treatment of UC can effectively protect siNLRP3 from degradation [41]. The use of material science technology can engineer exosomes. Martijn J W Evers et al. believed that the delivery of functional siRNA through extracellular vesicle-liposome hybrid nanoparticles combines the advantages of synthetic and biological drug delivery systems and may become a future therapeutic carrier [42]. The team of Carl M Schoellhamme found that low-frequency ultrasound for local delivery in the gastrointestinal tract can be used to deliver siRNA to the colonic mucosa of mice and knockdown the expression of target mRNA [43]. In addition, the Graph Neural Network model predicts that the circRNA-miRNA regulatory network has a very good effect [44], indicating that AI-driven circRNA function prediction has also become a new trend. DeepHeteroCDA constructs a multi-scale heterogeneous network integrating circRNA features and drug sensitivity profiles, employing Graph Convolutional Networks to predict circRNA-drug sensitivity associations [45]. Tanwei Xiong et al. utilized the Encyclopedia of RNA Interactomes (ENCORI) to predict and experimentally validate that DDX11-AS1 promotes LPS-induced intestinal mucosal cell apoptosis and inflammation by targeting miR-2355-5p [46]. Yichao Yan leveraged miRNet-constructed miRNA-mRNA networks to identify miR-200a-3p as a central regulator and identified pivotal hub genes in colorectal adenocarcinoma [47]. In this field of research, this interdisciplinary collaboration is promoting the research on the regulation of IEH based on ncRNAs towards precision and efficiency.

### The research hotspots and future directions of the regulation of IEH by ncRNAs

Changes in research hotspots are driven by a variety of factors. One of the key factors is technological advancement. With the development of sequencing technology, lncRNAs and circRNAs have become new hotspots for in-depth research. Clinical needs have also guided the research direction, and the increase in the incidence of IBD and other intestinal diseases worldwide in recent years has prompted scholars to increase the exploration of related mechanisms and therapeutic targets. At the same time, the strengthening of academic exchanges and cooperation also promotes the dissemination and proliferation of research hotspots, and the collision of ideas between different research teams has given rise to new research directions.

We summarized and predicted the research hotspots mainly through keyword analysis, in which the comprehensive analysis of keyword contribution network, clustering analysis and keywords with strongest citation bursts comprehensively demonstrated the academic community’s comprehensive exploration of the field over the past many years (Fig. 7). As a multifaceted concept, IEH has been systematically investigated at the cellular level through studies on intestinal epithelial cell proliferation, differentiation, apoptosis, migration, and tight junction (Fig. 7A). At the disease level, many scholars have investigated IEH dysfunction in different disease models, including IBS, UC, CD, sepsis, necrotizing small bowel colitis, cancer, intestinal ischemia/reperfusion injury, and celiac disease (Fig. 7A).

The keyword cluster analysis presented several relatively independent research themes, but the cluster groups did not exist in isolation from each other. For example, there are tight interactions between the gut microbiota, intestinal immunity, and intestinal permeability, and ncRNAs influence IEH by regulating these interactions.

#### Studying intestinal immunity and the gut microbiota from ncRNAs is a hot topic

Among the interactions between intestinal epithelial cells and the immune system, T cells, regulatory T cells, inflammation, tumor necrosis factor, extracellular vesicles, dendritic cells, and stem cells are research hotspots. In this complex immunoregulatory network, ncRNAs play a key role, especially miRNAs and lncRNAs, which act as sophisticated regulatory switches to finely regulate intestinal immune responses and inflammatory responses. Among them, miRNAs can affect intestinal immune response and intestinal inflammatory response by regulating the proliferation, differentiation and function of immune cells. For example, microRNA-219a-5p can suppress intestinal inflammation in IBD by inhibiting Th1/Th17-mediated immune responses [48]. Furthermore, it was shown that miR-221 and miR-222 regulate intestinal Th17 cell responses and are induced after IL-23 stimulation to limit the intensity of pro-inflammatory responses [49]. On the other hand, lncRNAs likewise play an integral role in several processes of intestinal immunity, including the regulation of apoptosis, lipid metabolism, and cell-to-cell interactions in the intestinal epithelium, which enhances the functional regulation of inflammation and regulatory T cells [50, 51]. Scholars have identified lncRNA IFNG-AS1 as a mediator in the inflammatory signaling cascade that regulates the balance between inflammation and anti-inflammatory cytokine production following T-cell stimulation [52]. The expression of Lnc-ITSN1-2 is increased in the intestinal mucosa of patients with IBD, and Lnc-ITSN1-2 promotes CD4 T-cell activation and proliferation in IBD and stimulates Th1/Th17 cell differentiation [53].

Non-coding RNAs not only have a significant effect on the regulatory level of immune cells, but also serve as a bridge in the relationship between gut microbes and the intestinal epithelial barrier. Several scholars have discussed the effect of ncRNAs on the intestinal epithelial barrier at the level of gut microbes and permeability (Fig. 7B). It is well known that there is a close interaction between ncRNAs and intestinal microbes. Non-coding RNAs can indirectly affect the nutrients available to gut microbes by regulating metabolic pathways in intestinal epithelial cells. Secondly, ncRNAs can also indirectly alter the metabolic properties of the gut flora by regulating the gut immune response to microbes. Several studies have shown that MiR-200b-3p improves the flora structure and increases the abundance of probiotics. At the same time, the abundance and activity of various gut flora can also affect ncRNAs, thereby maintaining IEH and influencing gut health through regulation. Researchers have found that flora can feed back into the intestinal microenvironment in the form of extracellular vesicles, thus suppressing the development of intestinal inflammation [54]. Intestinal flora can reprogram intestinal lipid metabolism by inhibiting the expression of lncRNA Snhg9 in small intestinal epithelial cells [55].

#### IBD has always been an important disease in the study of IEH

As can be seen from Fig. 7A, Tables 5 and 6, UC and CD have received a great deal of attention. It is well known that UC and CD, as the two main subtypes of IBD, have received extensive attention worldwide in recent years. Epidemiological data show that the incidence of IBD has shown a significant upward trend in the past two decades, especially in Asia, South America and other newly industrialized regions. The growth rate is particularly obvious, and in 2025 the global patients are expected to exceed 12 million [56]. The pathogenesis involves the multidimensional interactions of genetic susceptibility, dietary structural changes, immune dysregulation, and intestinal microbiota imbalance [57]. This complex pathological mechanism provides an important entry point for ncRNA research, as ncRNA can integrate multiple layers of pathogenic factors through epigenetic regulation [58].

Among them, miRNAs mainly target and bind to the 3’ UTR or other regions of mRNA through the seed sequence, recruit protein complexes to mediate translation inhibition or mRNA degradation, and affect gene expression [59]. LncRNAs and circRNAs form a specific regulatory network through multiple mechanisms. circRNAs and lncRNAs act as molecular sponges and competitively bind to miRNAs through the ceRNA mechanism to relieve their inhibitory effects. Some lncRNAs and circRNAs are involved in recruiting transcription factors or chromatin modification complexes to regulate gene transcription. In addition, specific lncRNAs and circRNAs can also exert translation regulatory functions by encoding functional peptides [60–63].

In Table 4, these 10 highly cited articles demonstrate significant credibility and academic value in the field of ncRNAs and IEH. Eight of these studies are closely related to IBD, exploring five distinct themes: (1) miRNA regulation of inflammatory signaling in IBD, (2) miRNA impacts on colitis-associated tumorigenesis, (3) identification of miRNAs associated with ileal and colonic CD, (4) lncRNA H19-mediated promotion of intestinal mucosal regeneration (5) novel insights into ncRNA-mediated epithelial barrier function. These 5 themes highlighted the critical roles of ncRNAs in intestinal inflammation and epithelial barrier, clarified the specific functions and mechanisms of various miRNAs in IBD, and provided a new perspective for understanding the pathogenesis of IBD. Meanwhile, the relationship between lncRNA H19 and mucosal regeneration was discovered, broadening the understanding of the role of lncRNA in intestinal diseases. Based on these findings, ncRNAs show enormous potential as both biomarkers and therapeutic targets. Modulating specific miRNA expression may improve intestinal inflammation and epithelial barrier repair. For example, miR31 reduces colonic epithelial inflammation by inhibiting inflammatory cytokine receptors (Il7R, Il17RA) and signaling proteins (GP130), while promoting epithelial regeneration through regulation of WNT and Hippo signaling pathways.

However, there are many limitations in the current research on ncRNAs and IBD. Differences between the mouse model of DSS-induced acute colitis and human IBD characterized by chronic recurrence may affect the translation of experimental results. In IBD, complex interactions between ncRNAs and with other signaling pathway networks have not been fully elucidated, and mechanistic resolution is incomplete. Moreover, most studies have focused on cellular experiments or animal models, but validation and application in humans require further research. In addition, how to solve the technical difficulties such as tissue-specific delivery and off-target effects still remains bottlenecks in clinical translational research.

#### The study of intestinal stem cell differentiation from the perspective of circRNA is a novel and significant research direction

Table 5 lists the IEH-associated miRNAs studied in 6 or more articles, while Table 6 presents IEH-associated lncRNAs studied in 3 or more publications. However, we found that no circRNAs have been studied more than twice in the literature. We counted the ncRNAs involved in the 667 IEH-associated articles, focusing on those that appeared miRNAs with 6 or more occurrences and lncRNAs with 3 or more occurrences. CircRNAs were not included in this section because no single circRNA was studied more than twice in the literature, which again indicated that miRNAs have been extensively studied by many scholars in the relationship between ncRNAs and IEH, whereas exploration of IEH from the perspective of lncRNAs, and especially circRNAs, remained limited. The lag in circRNA research primarily stems from technical bottlenecks and inadequate functional understanding. The sequence overlap between circRNAs and their homologous linear RNAs poses persistent technical challenges across detection, quantification, and functional characterization. Early researchers focused on the role of miRNAs, overlooking the functional diversity of circRNAs. Moreover, continuous advancements in sequencing technologies and bioinformatics have made research on circRNAs no longer challenging now. The effect of ISCs on IEH from the perspective of ncRNAs has not been sufficiently investigated. Therefore, we predict that studying ISC differentiation from the perspective of circRNAs could be novel and worthy of further exploration.

Firstly, the biological properties of circRNA fit the research needs of ISCs. The unique covalently closed circular structure of circRNA protects it from degradation by RNA exonucleases, and its half-life can reach more than 18 h, which is more than 4 times of the half-life of linear RNA [64]. The stability of circRNA provides a unique advantage for its long-term regulatory functions in ISCs. Then, circRNAs can regulate gene expression through various mechanisms such as miRNA sponge, RNA-binding protein interactions, and translational templates. CircPan3 binds to and promotes the stabilization of IL-13Rα1 mRNA and then activates the expression of Foxp1, which ultimately participates in the self-renewal of Lgr5^+^ ISCs [65]. In addition, the spatiotemporal-specific expression of circRNAs is a core feature of their involvement in ISC regulation. CircBtnl1 is significantly highly expressed in ISCs, while it is less expressed in the non-stem cell population. ISC regeneration is enhanced in circBtnl1 knockout mice at specific time points of radiation damage repair. CircBtnl1 inhibits self-renewal of ISCs under homeostatic conditions and regulates the molecular mechanisms of stemness maintenance by modulating Atf4/Sox9 signaling [66]. In addition, as shown in Fig. 8, exploring IEH through fecal ncRNAs has also emerged as a current research focus in the field. Colitis-associated cancers have become a research hotspot with significant translational significance and deserves further exploration in the future.

## Limitation

Although the study has made significant progress in the field of ncRNAs and IEH, several limitations remain. The data were primarily retrieved from English language literature in Web of Science, which may lead to certain biases. The citation data may underrepresent very recent high-impact papers due to time lag. No quality appraisal of individual studies was conducted. Despite these challenges, rigorous quality assessment of included literature was conducted, providing valuable insights into the current status and development trends of ncRNA regulation in IEH.

## Conclusion

The study presents the first detailed analysis of the major knowledge domains and emerging trends in ncRNA regulation of IEH, providing a comprehensive landscape of the current research status from multiple dimensions. Overall, the future development of ncRNA research in IEH is promising and full of opportunities and challenges. Future studies should establish multicenter clinical cohorts to validate fecal ncRNAs as non-invasive biomarkers for IBD and colorectal cancer. The deep integration of this field with artificial intelligence, bioinformatics, materials science and other disciplines will become a trend. International cooperation should be further deepened in order to integrate global resources to overcome problems and bring new breakthroughs in the diagnosis, treatment and prevention of intestinal diseases.

## Supplementary Information

Below is the link to the electronic supplementary material.

Supplementary Material 1

## Data Availability

The datasets analyzed during the current study are available from the corresponding author upon reasonable request.

## Abbreviations

IEC: intestinal epithelial cell
IBD: inflammatory bowel disease
UC: ulcerative colitis
CD: Crohn’s disease
NEC: Necrotizing Enterocolitis
CAC: colitis - associated cancer
HGHF: high glucose and high fat
intestinal I/R injury: intestinal ischemia-reperfusion (I/R) injury
IBS: irritable bowel syndrome
