# Supplementary material for: Global research trends in non-coding RNA and intestinal epithelial homeostasis: an integrated bibliometric analysis (2007–2024)
Source: Hereditas. 2025 Oct 17;162:213. doi: 10.1186/s41065-025-00560-y (PMC12535046; doi:10.1186/s41065-025-00560-y)
Supplement: Supplementary file 1 — Supplementary Material 1 [file 41065_2025_560_MOESM1_ESM.docx]

**Supplementary Materials**

The search content was set to “Query preview: (((((((ALL=(microRNA)) OR ALL=(miRNA)) OR ALL=(circular RNA)) OR ALL=(IncRNA)) OR ALL=(long non-coding RNA)) OR ALL=(circRNA)) OR ALL=(non-coding RNA)) AND (((((ALL=(intestinal)) OR ALL=(gut barrier dysfunction)) OR ALL=(gut epithelial function)) OR ALL=(gut barrier function)) OR ALL=(intestinal epithelial homeostasis)) OR ALL=(intestinal mucosal repair)) OR ALL=(intestinal mucosal injury))”.
